# Effects of Nitrogen Application Rates and Nitrogen Topdressing at Different Leaf Growth Stages on the Yield, Nitrogen Absorption, and Utilization of Nanjing 9108

**DOI:** 10.3390/plants15040668

**Published:** 2026-02-23

**Authors:** Zheshu Xu, Tao Li, Jingjing Cui, Jianghui Yu, Guangyan Li, Ying Zhu, Guodong Liu, Fangfu Xu, Qun Hu, Haiyan Wei

**Affiliations:** 1 Jiangsu Key Laboratory of Crop Genetics and Physiology, Jiangsu Key Laboratory of Crop Cultivation and Physiology, Agricultural College of Yangzhou University, Yangzhou 225009, China; 18361300936@163.com (Z.X.); 17673421385@163.com (T.L.); cjj19839371606@163.com (J.C.); ks179952@163.com (J.Y.); gyli@yzu.edu.cn (G.L.); 008396@yzu.edu.cn (Y.Z.); guodongliu@yzu.edu.cn (G.L.); xufangfu@yzu.edu.cn (F.X.); huqun@yzu.edu.cn (Q.H.); 2 Jiangsu Co-Innovation Center for Modern Production Technology of Grain Crops, Yangzhou University, Yangzhou 225009, China; 3 Research Institute of Rice Industrial Engineering Technology, Yangzhou University, Yangzhou 225009, China

**Keywords:** japonica rice, nitrogen regulation, yield, nitrogen use efficiency

## Abstract

The effects of nitrogen (N) application rates and N topdressing at different leaf growth stages on the yield, N absorption, and utilization of japonica rice cultivar Nanjing 9108 were studied to screen the optimal N management mode for high yield and high N use efficiency. A field experiment was conducted from 2023 to 2024, with nine N regulation treatments (94–351 kg ha^−1^) established through dynamic allocation of basal, tillering, and topdressing fertilizers. The results showed that with the increase of N application rate, the yield and N use efficiency of Nanjing 9108 first increased and then decreased. At a total N application rate of 270 kg ha^−1^, the N6 treatment (basal N + tiller N + topdressing at the 13th leaf stage) demonstrated optimal overall performance, achieving the highest yield and N use efficiency. Topdressing at the 13th leaf stage (coinciding with young panicle differentiation) promoted spikelet differentiation and large panicle formation, increasing grains per panicle by 2.36–2.20% compared to other treatments under the same N rate. The N6 treatment exhibited enhanced N uptake and utilization: N accumulation increased by 39.27–67.12% during the elongating to heading stage and by 7.14–62.24% during heading to maturity, while N apparent efficiency and agronomic efficiency rose by 3.51–14.68% and 29.22–58.25%, respectively. At heading, the proportion of high-effective leaf area in N6 was 1.52–7.05% higher than in N4, N5, and N7 treatments, accompanied by a slower leaf area decay rate. These traits provided sustained photosynthetic support for dry matter accumulation in mid-to-late growth stages. Consequently, dry matter accumulation in N6 increased by 5.85–33.44% (elongating to heading) and 0.42–26.98% (heading to maturity), leading to a yield advantage of 3.8–17.2% over other treatments. In summary, the N management strategy combining basal, tiller, and 13th-leaf topdressing at 270 kg ha^−1^ is most effective for achieving both high yield and high N efficiency in Nanjing 9108.

## 1. Introduction

Rice is one of the most important food crops in China, and its stable yield and quality improvement are directly related to national food security and residents’ dietary needs. With the upgrading of consumption, late-maturing medium japonica rice with both high yield potential and excellent eating quality has become the mainstream variety in the middle and lower reaches of the Yangtze River. Among them, Nanjing 9108 has become a major local cultivar due to its plump grains and excellent eating quality. Now its planting area has exceeded 52 × 10^4^ hectares in Jiangsu Province [1].

N is a core factor regulating rice growth and yield formation. Reasonable N application rate and period are crucial for increasing rice yield and N use efficiency [2]. Studies on japonica rice have shown that the peak period of N absorption is concentrated from elongating to heading stage. When the N application rate is within the range of 240–300 kg ha^−1^, rice has the potential to achieve high yield as well as improving the utilization of N fertilizer [3]. Either insufficient or excessive N supply is detrimental to obtaining a high yield of rice. Insufficient N supply limits the formation of effective tillers and spikelet differentiation, resulting in fewer panicles and spikelets per panicle. Excessive application of N not only fails to increase rice yield, but also leads to increased residual nitrate N in the soil and N_2_O emissions from the paddy fields [4].

In addition to controlling the total amount of N fertilizer, meticulous N fertilizer management at each critical stage of rice growth is also crucial for increasing rice yield and improving N fertilizer utilization efficiency [5,6]. Previous research has revealed that, an appropriate N fertilizer management strategy and increasing the proportion of spike fertilizer to 30–40% can reduce the occurrence of ineffective tillers. It can also promote the differentiation of rice spikelet and grain filling. As a result, the yield and quality of rice have been improved, and the N use efficiency has also been enhanced [7]. However, some studies using early-maturing late japonica rice varieties as experimental materials have shown that basal-tiller N and spike N each accounting for 50% of the total N application is a favorable ratio. Spike N should be applied at the 16th and 17th leaf stages, respectively. This N fertilizer management is conducive to obtaining sufficient panicles with high percentage of productive tillers and promoting the formation of large panicles with increased seed-setting rate, both of which contribute to high rice yield [8]. In the indica-japonica hybrid rice variety Yongyou 1538, the optimal ratio of basal, tiller, and spike fertilizer is 4∶2∶4 for high yield of rice. Compared to applying spike N fertilizer only at 16th leaf stage, splitting the application at the stages of 15th and 17th leaf stage, respectively, can increase rice yield more effectively. This approach optimizes the population’s high-effective leaf area and dry matter accumulation from heading to maturity, thereby achieving a better balance between high yield and good quality [9]. In addition, the fertilizer management mode with the ratio of basal, tiller, and spike fertilizer at 5:3:2 can optimize the dry matter accumulation and translocation efficiency of direct-seeded rice, enhance N fertilizer use efficiency, coordinate yield components, and ultimately achieve yield increase [10]. Additionally, studies have shown that when basal, tiller, and spike fertilizer are applied at 40%, 30%, and 20%, respectively, supplementing 10% grain fertilizer at the initiation of panicle development can continuously supply N, maintain photosynthetic function in the late growth stage, promote grain filling, and realize yield increase [11].

Now, extensive studies have been conducted on the effects of N management on yield, N absorption, and utilization of rice. However, the comprehensive mechanism of the coupling effect of different total N application rates and N topdressing at different leaf growth stages on the yield components, N absorption, and utilization of high yield, high effective, and good-tasting varieties is still unclear. This leads to the contradictions of “high yield but low efficiency” or “high efficiency but low yield” in production. Such contradictions are mainly attributed to the mismatch between N application schemes and varietal characteristics. In this study, Nanjing 9108 was used as the test material. Different N application rates and N topdressing at different leaf growth stage treatments were set to clarify the suitable N management mode for Nanjing 9108. This study aims to provide a scientific basis for improving its yield and N use efficiency, as well as a reference for varieties of the same type with the same total number of leaves on the main stem.

## 2. Results and Analysis

### 2.1. Effects of N Application Rates and N Topdressing at Different Leaf Growth Stages on the Yield

#### 2.1.1. Yield and Its Components

N application rate and N topdressing at different leaf growth stages had significant effects on rice yield and its components. Compared with the rice yield of N1 treatment, rice yields of N2 and N3 treatments increased by 15.74% and 19.54%, respectively (Table 1). Among all treatments receiving 270 kg ha^−1^ (N4, N5, N6, and N7) N fertilizer, the N6 treatment achieved the highest yield. Compared with the rice yield of N6 treatment, rice yields of N8 and N9 treatments decreased by 6.18% and 8.73% on average, respectively, despite their additional N fertilizer application. The appropriate number of panicles and spikelets per panicle, which form a sufficient spikelet number of rice population, combined with a high seed-setting rate and well-filled grains, are the main reasons for the highest yield of N6 treatment.

#### 2.1.2. Tiller Dynamics

At maturity, the tiller number of rice increased with the increase of N application rate and the treatments of N8 and N9 had the largest tiller number (Table 2). Among all treatments receiving 270 kg ha^−1^ (N4, N5, N6, and N7) N fertilizer, the tiller number of rice at each growth stage decreased with the delay of N application, and the N4 treatment showed the highest tiller number. The N6 treatment achieved the maximum value in the percentage of productive tillers, which was 1.43–7.98% higher than those of N4, N5, and N7 treatments. Compared with the N6 treatment, when N fertilizer application was increased to 351 kg ha^−1^, the percentage of productive tillers did not increase significantly.

#### 2.1.3. Leaf Area Index and Leaf Area Decay Rate

Among all treatments receiving 270 kg ha^−1^ (N4, N5, N6, and N7) N fertilizer, leaf area index at the elongating stage and the decline rate of leaf area decreased with the delay of N application (Table 3). The N6 treatment showed the greatest effective leaf area index and the percentage of high effective leaf area at the heading stage, as well as the leaf area index at the maturity, which were 1.34–10.56%, 1.52–7.05%, and 3.35–31.21% higher than those of N4, N5, and N7 treatments. Compared with N6 treatment, the percentages of high effective leaf area of N8 and N9 treatments were decreased by 5.00% and 5.45% on average, respectively.

#### 2.1.4. Dry Matter Accumulation

The dry matter accumulation of rice increased with the application of N fertilizer and reached the maximum under 351 kg ha^−1^ N application (Table 4). Among all treatments receiving 270 kg ha^−1^ (N4, N5, N6, and N7) N fertilizer, rice dry matter accumulation at key growth stages initially increased with the delay of N application, peaked in the N6 treatment, and subsequently decreased. Compared with N6 treatment, the harvest index of N8 and N9 treatments was reduced by 2.52% and 4.76% on average, respectively.

#### 2.1.5. Dry Matter Accumulation and Its Proportion at Different Growth Stages

The dry matter accumulation before elongating and during elongating to the heading stage reaches its maximum under 351 kg ha^−1^ N application (Table 5). Among all treatments receiving 270 kg ha^−1^ (N4, N5, N6, and N7) N fertilizer, the N6 treatment had the highest accumulation of dry matter during the stages from elongating to heading and from heading to maturity. Compared with the N4, N5, and N7 treatments, the dry matter accumulation in these two stages increased by 6.76–30.32% and 2.43–22.82%, respectively. When the N fertilizer application rate was increased to 351 kg ha^−1^, the proportion of dry matter accumulation increased from elongating to the heading stage but decreased from heading to the maturity stage.

### 2.2. Effects of N Application Rates and N Topdressing at Different Leaf Growth Stages on the N Absorption and Utilization

#### 2.2.1. N Concentration and N Accumulation

At each growth stage, the N concentration increased with the increase of N application rate. The N concentration was the highest when the N application rate was 351 kg ha^−1^ (Table 6). Among all treatments receiving 270 kg ha^−1^ (N4, N5, N6, and N7) N fertilizer, the N concentration at maturity initially increased with the delay of N application, peaked in the N6 treatment, which was 1.76–4.53% higher than that of N4, N5, and N7 treatments. The trend of N accumulation was similar to the trend of N concentration at each stage. At maturity, compared with N6 treatment, the average N accumulation of N8 and N9 treatments was significantly increased by 6.51% and 6.06%, respectively.

#### 2.2.2. N Accumulation and Its Proportion at Different Growth Stages

Before elongating, N accumulation was significantly higher in the N4, N5, N8, and N9 treatments compared to the others (Table 7). Among all treatments receiving 270 kg ha^−1^ (N4, N5, N6, and N7) N fertilizer, N accumulation during the elongating to heading and heading to maturity stages initially increased with delayed N application and peaked in the N6 treatment. Compared individually to the N4, N5, and N7 treatments, the N accumulation in the N6 treatment was 6.71–76.83% higher during the elongating to heading stage, and 12.43–59.76% higher during the heading to maturity stage. At the higher N application rate of 351 kg ha^−1^ (N8 and N9 treatments), the proportion of N accumulated during the heading to maturity stage was significantly reduced by 31.93% and 26.58%, respectively, compared to the N6 treatment.

#### 2.2.3. N Translocation

When the N fertilizer application rate reached or exceeded 270 kg ha^−1^, NTA in rice leaves and stems increased significantly (Table 8). Among all treatments receiving 270 kg ha^−1^ (N4, N5, N6, and N7) N fertilizer, the NTA in both leaves and stems initially increased with the delay of N application and peaked in the N6 treatment. Compared individually to the N4, N5, and N7 treatments, the NTA in the N6 treatment was 3.95–6.55% higher in leaves and 10.65–15.30% higher in stems. However, NTAR and NTCR in leaves and stems gradually decreased with the delay of N application rate and N4 treatment had the maximum value. Compared with N5, N6, and N7 treatments, the N4 treatment showed 1.10–4.31% and 1.09–4.15% higher NTAR in leaves and stems. Correspondingly, its NTCR of leaf and stem were 1.57–5.37% and 0.91–3.04% higher than the three treatments, respectively. In terms of the N increment in panicle and hull, N6 treatment was the highest. Notably, when N application was increased to 351 kg ha^−1^ (N8, N9), the N increment in panicle and hull decreased by 13.22% and 18.30% on average compared with N6 treatment, respectively.

#### 2.2.4. N Use Efficiency

With the increase in N application rate, NPE and NPFP gradually decreased (Table 9). Among all treatments receiving 270 kg ha^−1^ (N4, N5, N6, and N7) N fertilizer, N use efficiency initially increased with the delay of N application, peaked in the N6 treatment, and subsequently decreased. Compared with the N4, N5, and N7 treatments, NAPE, NPE, NAGE, and NPFP of N6 treatment increased by 9.07–38.18%, 4.53–15.57%, 13.90–59.69%, and 5.03–17.26%, respectively. When N application increased to 351 kg ha^−1^ (N8, N9), the NAPE, NPE, NAGE, and NPFP were significantly decreased compared with the N6 treatment, while the NRG increased significantly by 10.81% and 13.51%.

## 3. Discussion

### 3.1. Effects of N Application Rates and N Topdressing at Different Leaf Growth Stages on the Yield of Nanjing 9108

The coordination of yield components is the core of high rice yield, and N fertilizer is a key factor regulating the balance between panicle number, spikelets per panicle, seed-setting rate, and 1000-grain weight [12]. This study found that the N6 treatment (total N application rate of 270 kg ha^−1^, combined application of basal N, tiller N, and N topdressing at the stage of 13th leaf emergence) achieved the highest yield by regulating N application rates and N topdressing at different leaf age stages.

Compared with the low N treatments (N1, N2, N3), the N6 treatment, through the split and reasonable application of basal N, tiller N, and N topdressing at the stage of 13th leaf emergence, not only promoted the occurrence of effective tillers and stabilized panicle number in the early stage but also facilitated the formation of large panicles [13]. Compared with other treatments under the same N application rate of 270 kg ha^−1^, the N6 treatment applied N at the stage of 13th leaf emergence (young panicle differentiation stage). N applied at this period can increase the ratio of cytokinin to auxin in panicles, and is conducive to extending the differentiation period and promoting spikelet differentiation, thus increasing the number of differentiated spikelets per panicle. This laid a pre-requisite for achieving large panicles with more grains [14]. Meanwhile, N application at this stage is synchronized with the growth of the top three leaves, which can promote chlorophyll synthesis and photosynthetic enzyme activity in leaves. The results showed that the N6 treatment had a low decreasing rate of leaf area, indicating slow leaf senescence in the late stage. N application at the stage of 4th leaf emergence from the top can extend the leaf functional period and continuously provide nutrients for grain filling [15]. The suitable tiller number and efficient leaf area of the N6 treatment resulted in higher dry matter accumulation during elongating to heading and heading to maturity stages, as well as a higher percentage of productive tillers, which further improved the seed-setting rate and 1000-grain weight, ultimately achieving high yield [16]. Compared with the high N treatments (N8, N9), although the N6 treatment had a lower N application rate, the proportion of N applied in the early, middle, and late stages was reasonable, resulting in fewer ineffective tillers and a suitable tiller population number. At the same time, it had a higher average proportion of high-effective leaf area at the heading stage, which could efficiently convert light energy into carbohydrates. Additionally, the reasonable leaf area index at the heading and maturity stages ensured good ventilation and light transmission of the population, promoting the translocation of carbohydrates to grains, thereby improving the seed-setting rate and 1000-grain weight [17].

### 3.2. Effects of N Application Rates and N Topdressing at Different Leaf Growth Stages on N Absorption and Utilization of Nanjing 9108

N is the most demanding essential nutrient during rice growth and development, and the efficiency of N absorption and utilization directly determines the final yield formation of rice [18]. Japonica rice has a fast N absorption rate and large N absorption capacity after elongating. Implementing “precise postponement of N application” and appropriately increasing the proportion of spike fertilizer can meet the N demand of japonica rice in the middle and late growth stages, thereby improving rice yield and N absorption and utilization efficiency [19]. The results showed that the N6 treatment had the highest N use efficiency.

Compared with the low N treatments (N1, N2, N3), the N6 treatment had significantly higher NAPE and NAGE, but relatively lower NPE and NPFP. This phenomenon is not due to insufficient N use capacity but to differences in N supply levels. Under N-deficient conditions, N is preferentially translocated from vegetative organs (leaves and stems) to panicles, rather than reflecting high N use efficiency [20]. In contrast, through the split and reasonable application of basal N, tiller N, and N topdressing at the stage of 13th leaf emergence, the N6 treatment not only provided a N foundation for the occurrence of effective tillers in the early stage but also maintained a high N concentration at the heading and maturity stages through N application at the stage of 13th leaf emergence. Meanwhile, it reduced the decreasing rate of leaf area, extended the leaf functional period, and ensured the continuous accumulation of photosynthates, providing material support for the efficient conversion of N to grains [21,22]. Under the same N application rate of 270 kg ha^−1^ with other treatments, the N6 treatment had the highest matching degree between N application period and N absorption demand, as well as its N demand peak during the growth period of Nanjing 9108. The N6 treatment applied N at the stage of 13th leaf emergence led to significantly higher N concentration at the heading and maturity stages, and higher N accumulation during the elongating to heading and heading to maturity stages compared with other treatments under the same N application rate of 270 kg ha^−1^, which coincides with the critical period of young panicle differentiation. The absorbed N can directly participate in physiological processes such as spikelet primordium differentiation and panicle axis elongation [23,24], significantly increasing the spikelets per panicle. This avoids the problems of early N application (N4, N5 treatments) leading to N consumption by ineffective tillers in the early stage, and late N application (N7 treatment) missing the critical period of spikelet differentiation, resulting in insufficient spikelet number and more N remaining in leaves and stems [25]. The high N concentration of the N6 treatment endowed leaves with higher chlorophyll content and photosynthetic enzyme activity, maintaining longer photosynthetic accumulation and providing an energy foundation for N absorption [26]. The N accumulation in panicles at maturity partially comes from N accumulation after heading and partially from N translocation from leaves and stems [27], both are significantly positively correlated with rice yield [28]. The N translocation amount in leaves and stems of the N6 treatment was significantly higher than that of N4, N5, and N7 treatments (Table 9). Moreover, N application at the young panicle differentiation stage can enhance the material transport efficiency from stems to panicles, thereby promoting grain filling and forming high yield. This further indicates that N in this treatment not only “is sufficiently absorbed” but also “is well translocated” [29]. Compared with the high N treatments (N8, N9), the N6 treatment had slightly lower N concentration and N accumulation at each stage, but showed outstanding advantages in N use efficiency. Although the N8 and N9 treatments increased N accumulation by increasing N application rate, the N was mostly used for redundant growth of vegetative organs, leading to decreased population ventilation and light transmission, reduced leaf photosynthetic efficiency, and insufficient grain filling [6].

## 4. Materials and Methods

### 4.1. Experimental Location and Test Variety

**Figure 1:**
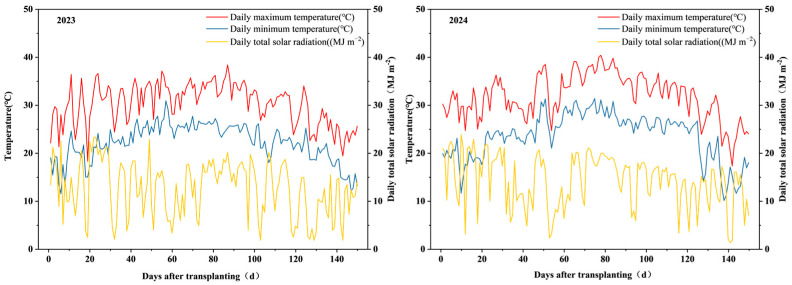
Daily maximum, minimum temperature and total solar radiation during the entire rice-growing seasons in 2023 and 2024.

Field experiments were conducted at Chenxing Village (32°32′ N, 119°55′ E), Yangzhou City, in 2023 and 2024. The meteorological conditions during the rice growing seasons of 2023 and 2024 were generally suitable, with no extreme temperature or radiation events that adversely affected rice growth and development. The dynamic changes of daily maximum/minimum temperature and total solar radiation were basically consistent between the two years, conforming to the typical climatic characteristics of rice cultivation in Yangzhou. Meteorological data for the experimental site during the rice-growing periods were provided by the National Meteorological Science Data Center (Figure 1). The field soil was sandy loam with good and balanced fertility. Soil organic matter was determined by the total organic carbon (TOC) analyzer method using a multi N/C 3100 TOC analyzer (Analytikjena, Gena, Germany) [30]. The soil organic matter concentration was 31.52 g/kg, alkali-hydrolyzable N concentration was 198.1 mg/kg, available P concentration was 31.6 mg/kg, and available K concentration was 210 mg/kg. The experimental variety was late-maturing medium japonica rice Nanjing 9108, which has six elongated internodes, 16 total leaves on the main stem. Its whole growth duration is about 150 days.

### 4.2. Experimental Design

Pre-germinated seeds were sown into seedbeds on 19 March 2023 and 18 March 2024, respectively. The row and plant spacing was set at 30 cm × 12 cm, with four to five seedlings transplanted per hill. Field experimental were arranged in a randomized complete block design with 10 N treatments. The experiment was replicated three times, and each plot area was 75 m^2^. The plots were separated by a ridge that was wrapped with plastic film, and each plot was irrigated or drained independently. The rationale for the specific nitrogen application rates (94–351 kg ha^−1^) and their distribution across growth stages was based on our previous field observations and key developmental stages of the late-maturing medium japonica rice Nanjing 9108 (Table 10). Fertilization at the 9th leaf growth age promotes the increase of effective tillers, while application at the 11th leaf growth stage tends to stimulate ineffective tillering. The 13th leaf growth stage coincides with the spikelet differentiation stage, during which nitrogen application enhances spikelet formation. The 15th leaf growth stage corresponds to pollen formation, a critical phase for grain development. Basal fertilizer was applied 1 day before transplanting. Tiller fertilizer was applied 7 days after transplanting. Topdressing N fertilizer in the middle and late stages of each treatment was applied when the leaf of the corresponding leaf fully emerged. In addition, 135 kg ha^−1^ P (as superphosphate) and 270 kg ha^−1^ K (as KCl) were also applied. Field water management, pest control, and other agronomic cultivation measures were carried out in accordance with high-yield cultivation standards.

### 4.3. Sampling and Data Collection

#### 4.3.1. Yield and Yield Components

At maturity, 50 random hills per treatment were sampled for effective panicle number. Five of them were used to determine grains per panicle, seed-setting rate, and 1000-grain weight. Yield was measured from 100 hills after threshing, impurity removal, and airdrying, adjusted to 14.5% moisture.

#### 4.3.2. Tiller Dynamics

Post-seedling regreening, one observation point per plot was set. Ten random hills per point were sampled to record tiller number at elongation, heading, and maturity.

#### 4.3.3. Leaf Area Index (LAI)

At the elongating, heading, and maturity stages, three hills per plot were sampled. Leaf area was measured with a portable leaf area meter (Li-3000A, LI-COR, Lincoln, NE, USA).

#### 4.3.4. Dry Matter Accumulation

At the elongating, heading, and maturity stages, five hills per plot were sampled, among which two hills were used as whole samples, and three hills were divided into organs (leaves and stems before heading; leaves, stems, and panicles after heading). Samples were oven-killed (105 °C, 30 min) then dried (80 °C to constant weight) for dry weight determination of whole plants and organs.

#### 4.3.5. N Concentration

Plant and organ samples (above stages) were ground and sieved (80-mesh). N concentration was determined by Kjeldahl method and used an automatic Kjeldahl N analyzer (Kjeltec 8400, Foss Tecator, Hillerod, Denmark) [31], with N use efficiency-related indices calculated simultaneously.

#### 4.3.6. Data Calculation and Statistical Methods

Microsoft Excel 2016 and SPSS 27.0 were used for data processing and correlation analysis.

Percentage of productive tillers (%) = Tillers number at maturity stage/Tillers number at elongating stage × 100;

Decreasing rate of leaf area (LAI d^−1^) = (Effective leaf area index at heading stage − Effective leaf area index at maturity stage)/Growth days from heading to maturity;

N absorption (NA, kg ha^−1^) = Above-ground dry weight at that stage × N concentration;

Stage N accumulation (kg ha^−1^) = N accumulation in the above-ground part at the later growth stage − N accumulation in the above-ground part at the previous growth stage;

Leaf (stem) N translocation amount (NTA, kg ha^−1^) = N accumulation in leaves (stem) at heading stage − N accumulation in leaves (stem) at maturity stage;

Apparent leaf (stem) N translocation rate (NTAR, %) = Leaf (stem) N translocation amount/N accumulation in leaves (stem) at heading stage × 100;

Leaf (stem) N translocation contribution rate (NTCR, %) = Leaf (stem) N translocation amount/N accumulation in panicles at maturity stage × 100.

N apparent efficiency (NAPE, %) = (Total N accumulation of plants in N-applied plots − Total N accumulation of plants in N free plot)/N application rate × 100;

N physiological efficiency (NPE, kg kg^−1^) = (Rice yield in N-applied plots − Rice yield in N free plot)/(Total N accumulation of plants in N-applied plots − Total N accumulation of plants in N free plot);

N agronomic efficiency (NAGE, kg kg^−1^) = (Rice yield in N-applied plots − Rice yield in N free plot)/N application rate;

N partial factor productivity (NPFP, kg kg^−1^) = Grain yield/N application rate;

N requirement for 100 kg grains (NRG, kg kg^−1^) = Total N accumulation of plants/Grain yield × 100.

## 5. Conclusions

N application rates and N topdressing at different leaf growth stages play a key regulatory role in the yield formation, N absorption, and utilization of Nanjing 9108. With the increase of N application rate, the yield and N use efficiency of this variety initially increased and peaked at N application rate of 270 kg ha^−1^, which achieved a balance between high yield and high N efficiency. Under this N application rate, the mode of “basal N + tiller N + N topdressing at the stage of 13th leaf emergence” performed the best. The combined application of basal N and tiller N in the early stage coordinately promoted the occurrence of effective tillers, resulting in a suitable population tiller number and high percentage of productive tillers. N application at the stage of 13th leaf emergence (young panicle differentiation stage) could effectively promote spikelet differentiation, increase the number of grains per panicle, and improve N absorption and utilization during key growth stages. The absorbed N significantly promoted the growth of above-ground parts, enlarged the high-effective photosynthetic leaf area at the heading stage, and reduced the decreasing rate of leaf area, thereby providing sufficient photosynthates for dry matter accumulation, and promoting the formation of large panicles and plump grains. Ultimately, the synergistic unification of high yield and high N use efficiency of Nanjing 9108 was achieved.

## Figures and Tables

**Table 1:** Effects of different N regulations on rice yield and yield components.

Treatment	No. of Panicles (×10^4^ ha^−1^)		Spikelets Per Panicle		Seed-Setting Rate (%)		1000-Grain Weight (g)		Grain Yield (t ha^−1^)	
Year	2023	2024	2023	2024	2023	2024	2023	2024	2023	2024
N1	260.25 g	257.73 e	116.1 f	116.4 fg	95.9 a	95.0 ab	25.2 a	25.2 a	7.03 h	7.20 h
N2	287.25 f	284.86 d	132.0 c	132.0 c	95.1 b	93.8 bc	24.4 bc	24.4 c	8.19 g	8.28 g
N3	310.80 e	307.46 cd	123.5 e	123.2 de	94.8 b	93.5 c	25.2 a	25.3 a	8.48 f	8.53 f
N4	362.25 b	356.81 ab	117.5 f	118.3 ef	92.6 ef	91.1 ef	24.2 c	24.1 c	9.02 e	9.07 e
N5	334.95 c	331.84 bc	128.0 d	128.4 cd	93.0 de	91.4 ef	24.8 ab	24.8 b	9.45 d	9.54 d
N6	326.10 cd	324.96 c	141.5 a	141.9 a	94.4 bc	92.8 cd	24.9 ab	24.9 b	10.57 a	10.63 a
N7	320.55 de	316.49 c	138.5 b	138.3 ab	93.7 cd	92.1 de	25.2 a	25.2 a	10.18 b	10.00 b
N8	377.10 a	375.19 a	129.0 d	129.2 c	91.8 f	90.7 f	23.3 d	23.3 d	10.07 b	9.82 c
N9	354.30 b	352.58 ab	133.5 c	133.2 bc	92.3 ef	91.0 ef	23.3 d	23.3 d	9.75 c	9.60 d

Different lowercase letters following data within the same column indicate significant differences among treatments at the 5% probability level (*p* < 0.05). The same convention applies to subsequent tables.

**Table 2:** Effects of different N regulations on tiller dynamics and the percentage of productive tillers.

Treatment	Tillers Number (×10^4^ ha^−1^)						Percentage of Productive Tillers (%)	
	Elongating		Heading		Maturity			
Year	2023	2024	2023	2024	2023	2024	2023	2024
N1	339.00 d	336.05 e	306.60 d	300.58 d	260.25 g	257.73 e	76.86 b	76.97 b
N2	344.55 d	345.45 e	310.35 d	307.95 d	287.25 f	284.86 d	83.37 a	82.73 cd
N3	427.95 c	424.93 d	372.15 c	368.32 c	310.80 e	307.46 cd	72.63 cd	72.41 a
N4	521.10 a	519.53 a	436.20 a	431.93 ab	362.25 b	356.81 ab	69.53 e	68.70 e
N5	460.05 b	462.07 bc	404.85 b	397.38 bc	334.95 c	331.84 bc	72.83 cd	71.94 cd
N6	432.15 c	431.73 cd	381.00 c	374.7 c	326.10 cd	324.96 c	75.46 b	75.45 b
N7	429.45 c	427.76 d	375.90 c	367.85 c	320.55 de	316.49 c	74.67 bc	74.08 bc
N8	533.55 a	521.10 a	444.75 a	439.37 a	377.10 a	375.19 a	70.70 de	72.01 de
N9	464.10 b	467.24 b	410.85 b	400.94 bc	354.30 b	352.58 ab	76.35 b	75.43 b

Different lowercase letters following data within the same column indicate significant differences among treatments at the 5% probability level (*p* < 0.05).

**Table 3:** Effects of different N regulations on leaf area index and the decreasing rate of leaf area.

Treatment	Elongating		Heading				Maturity		Decreasing Rate of Leaf Area (LAI d^−1^)	
			Effective Leaf Area Index		Percentage of High Effective Leaf Area (%)					
**Year**	**2023**	**2024**	**2023**	**2024**	**2023**	**2024**	**2023**	**2024**	**2023**	**2024**
N1	2.72 c	2.59 d	4.05 f	4.11 f	68.97 g	68.91 g	1.41 g	1.29 g	0.0507 g	0.0564 e
N2	2.72 c	2.70 d	4.90 e	4.76 e	69.12 g	69.03 g	1.75 f	1.75 f	0.0606 e	0.0603 d
N3	3.19 b	3.09 c	4.91 e	4.81 e	69.89 f	69.82 f	1.99 e	2.08 e	0.0562 f	0.0545 e
N4	3.96 a	3.92 a	5.81 d	5.84 d	69.98 f	69.93 f	2.11 e	2.12 e	0.0727 c	0.0745 bc
N5	3.69 a	3.62 b	6.13 cd	6.18 cd	71.75 c	71.66c	2.44 d	2.48 d	0.0724 c	0.0740 c
N6	3.21 b	3.17 c	6.42 bc	6.46 c	74.90 a	74.87 a	2.77 bc	2.78 c	0.0717 cd	0.0736 c
N7	3.19 b	3.13 c	6.35 c	6.36 c	73.84 b	73.69 b	2.65 c	2.72 c	0.0712 d	0.0728 c
N8	3.98 a	3.94 a	7.37 a	7.43 a	71.21 cd	71.07 cd	3.34 a	3.44 a	0.0807 a	0.0798 a
N9	3.68 a	3.64 b	6.87 b	6.86 b	70.82 de	70.79 de	2.94 b	2.96 b	0.0785 b	0.0780 ab

Different lowercase letters following data within the same column indicate significant differences among treatments at the 5% probability level (*p* < 0.05).

**Table 4:** Effects of different N regulations on dry matter accumulation of rice.

Treatment	Dry Matter Accumulation (t/ha^−1^)						Harvest Index (%)	
	Elongating		Heading		Maturity			
Year	2023	2024	2023	2024	2023	2024	2023	2024
N1	3.16 c	3.18 e	7.89 g	7.92 h	12.71 h	13.01 h	55.31 a	55.93 a
N2	3.16 c	3.29 d	9.13 f	9.13 g	14.76 g	14.82 g	55.47 a	55.32 a
N3	3.92 b	3.92 c	9.94 e	9.95 f	15.81 f	15.80 f	53.65 b	55.88 a
N4	4.55 a	4.57 a	11.07 d	11.26 e	17.19 e	17.19 e	52.47 b	53.99 b
N5	4.12 b	4.18 b	11.48 cd	11.51 e	17.97 d	17.95 d	52.56 b	52.74 bc
N6	3.92 b	3.91 c	12.62 b	12.42 c	19.87 b	19.95 b	53.19 b	53.14 bc
N7	3.92 b	3.90 c	12.00 c	11.94 d	19.23 c	19.14 c	52.97 b	53.28 bc
N8	4.55 a	4.56 a	13.74 a	13.54 a	20.54 a	20.45 a	49.05 c	52.25 c
N9	4.12 b	4.21 b	13.09 b	13.03 b	19.98 b	20.04 b	48.80 c	48.02 d

Different lowercase letters following data within the same column indicate significant differences among treatments at the 5% probability level (*p* < 0.05).

**Table 5:** Effects of different N regulations on dry matter accumulation and its proportion.

Treatment	Before Elongating				Elongating to Heading				Heading to Maturity			
	Biomass (t ha^−1^)		Ratio (%)		Biomass (t ha^−1^)		Ratio (%)		Biomass (t ha^−1^)		Ratio (%)	
Year	2023	2024	2023	2024	2023	2024	2023	2024	2023	2024	2023	2024
N1	3.16 c	3.18 e	24.84 b	24.41 b	4.74 g	4.74 g	37.26 e	36.44 e	4.82 f	5.09 e	37.90 a	39.15 ab
N2	3.16 c	3.29 d	21.38 de	22.23 d	5.97 f	5.84 f	40.45 c	39.38 cd	5.64 e	5.69 d	38.18 a	38.39 bc
N3	3.92 b	3.92 c	24.81 b	24.83 b	6.02 f	6.03 f	38.08 de	38.16 de	5.87 de	5.85 d	37.12 abc	37.01 bcd
N4	4.55 a	4.57 a	26.47 a	26.58 a	6.52 e	6.69 e	37.96 de	38.92 cd	6.11 d	5.93 d	35.58 d	34.49 de
N5	4.12 b	4.18 b	22.95 c	23.29 c	7.34 d	7.33 d	40.86 c	40.84 bc	6.50 c	6.44 c	36.19 cd	35.88 cde
N6	3.92 b	3.91 c	19.73 f	19.60 f	8.70 b	8.51 b	43.79 ab	42.66 ab	7.25 a	7.53 a	36.48 bcd	37.74 bc
N7	3.92 b	3.90 c	20.40 ef	20.38 e	8.08 c	8.04 c	42.04 bc	41.99 ab	7.22 a	7.21 ab	37.56 ab	37.63 bc
N8	4.55 a	4.56 a	22.15 cd	22.27 d	9.20 a	8.98 a	44.79 a	43.94 a	6.79 bc	6.91 bc	33.06 f	33.79 e
N9	4.12 b	4.21 b	20.65 ef	21.01 e	8.96 ab	8.82 ab	44.86 a	44.05 a	6.89 ab	7.01 b	34.50 e	34.95 de

Different lowercase letters following data within the same column indicate significant differences among treatments at the 5% probability level (*p* < 0.05).

**Table 6:** Effects of different N regulations on N concentration and N accumulation of rice.

Treatment	N concentration (%)						N accumulation (kg ha^−1^)					
	Elongating		Heading		Maturity		Elongating		Heading		Maturity	
Year	2023	2024	2023	2024	2023	2024	2023	2024	2023	2024	2023	2024
N0	1.66 e	1.54 e	0.97 f	0.96 f	0.78 f	0.76 h	41.10 d	36.88 d	67.20 h	67.17 h	86.85 i	88.39 i
N1	1.84 d	1.72 d	1.05 e	1.05 e	0.84 e	0.84 g	58.20 c	54.68 c	82.95 g	82.97 g	106.95 h	109.33 h
N2	1.84 d	1.72 d	1.21 d	1.22 d	1.00 d	1.02 f	58.20 c	56.67 c	110.25 f	110.93 f	148.05 g	151.15 g
N3	2.05 c	2.03 c	1.20 d	1.23 d	1.00 d	1.02 f	80.40 b	79.45 b	118.95 e	122.39 e	158.40 f	161.16 f
N4	2.28 b	2.19 b	1.43 c	1.43 c	1.10 c	1.11 e	103.65 a	100.08 a	158.55 d	160.97 d	189.75 e	190.78 e
N5	2.49 a	2.38 a	1.43 c	1.44 c	1.13 bc	1.14 cd	102.60 a	99.48 a	163.65 d	165.73 d	204.00 d	204.64 d
N6	2.05 c	1.97 c	1.44 c	1.45 c	1.15 bc	1.16 c	80.40 b	77.03 b	181.20 b	180.10 b	228.60 b	230.41 b
N7	2.05 c	1.97 c	1.46 c	1.46 c	1.14 bc	1.13 d	80.40 b	76.81 b	174.90 c	173.66 c	219.15 c	216.29 c
N8	2.28 b	2.19 b	1.53 b	1.54 b	1.20 ab	1.19 b	103.65 a	99.77 a	209.70 a	208.51 a	246.60 a	242.32 a
N9	2.49 a	2.38 a	1.57 a	1.58 a	1.23 a	1.21 a	102.60 a	100.04 a	204.90 a	205.84 a	245.25 a	241.57 a

Different lowercase letters following data within the same column indicate significant differences among treatments at the 5% probability level (*p* < 0.05).

**Table 7:** Effects of different N regulations on stage N accumulation and its proportion.

Treatment	Before Elongating				Elongating to Heading				Heading to Maturity			
	NA (t ha^−1^)		Ratio (%)		NA (t ha^−1^)		Ratio (%)		NA (t ha^−1^)		Ratio (%)	
Year	2023	2024	2023	2024	2023	2024	2023	2024	2023	2024	2023	2024
N0	41.10 d	36.88 d	47.38 b	41.74 c	26.10 f	30.29 f	30.04 d	34.26 b	19.65 e	21.22 f	22.57 b	23.99 ab
N1	58.20 c	54.68 c	54.37 a	49.99 b	24.75 f	28.29 f	23.17 f	25.86 c	24.00 e	26.35 ef	24.48 a	24.14 ab
N2	58.20 c	56.67 c	39.29 cd	37.49 b	52.05 d	54.27 d	35.18 c	35.90 b	37.80 c	40.22 bc	25.54 a	26.61 a
N3	80.40 b	79.45 b	50.74 ab	49.29 b	38.70 e	42.93 e	24.40 f	26.67 c	39.30 bc	38.78 bc	24.86 a	24.04 ab
N4	103.65 a	100.08 a	54.64 a	52.47 a	54.75 cd	60.89 cd	27.88 e	31.92 b	31.35 d	29.81 de	16.49 e	15.62 de
N5	102.60 a	99.48 a	50.34 b	48.63 b	61.05 c	66.26 c	29.91 d	32.37 b	40.35 bc	38.90 bc	19.76 d	19.01 cd
N6	80.40 b	77.03 b	35.15 e	33.43 e	100.95 ab	103.07 ab	44.15 a	44.73 a	47.25 a	50.32 a	20.70 c	21.84 bc
N7	80.40 b	76.81 b	36.67 de	35.51 d	94.35 b	96.85 b	43.12 ab	44.79 a	44.25 ab	42.62 ab	20.21 cd	19.70 c
N8	103.65 a	99.77 a	42.05 c	41.17 c	106.05 a	108.74 a	42.99 ab	44.90 a	36.90 c	33.81 c	14.97 f	13.94 e
N9	102.60 a	100.04 a	41.86 c	41.44 c	102.15 a	105.80 a	41.63 b	43.90 a	40.50 bc	35.72 bc	16.51 e	14.65 e

NA: N accumulation. Different lowercase letters following data within the same column indicate significant differences among treatments at the 5% probability level (*p* < 0.05).

**Table 8:** Effects of different N regulations on N translocation characteristics of various plant organs.

Treatment	NTA (kg ha^−1^)				NTAR (%)				NTCR (%)				NIPH (kg ha^−1^)	
	Leaf		Stem		Leaf		Stem		Leaf		Stem			
Year	2023	2024	2023	2024	2023	2024	2023	2024	2023	2024	2023	2024	2023	2024
N0	21.60 e	19.30 f	12.45 f	14.04 g	64.11 a	64.49 a	55.41 a	53.75 a	37.40 a	32.24 cd	21.66 d	23.47 d	48.30 h	49.02 h
N1	25.80 d	22.00 e	15.00 e	17.39 f	59.78 d	60.35 c	54.36 b	52.56 a	37.17 a	31.31 de	22.35 d	24.71 cd	55.80 g	56.82 g
N2	28.80 c	25.72 d	21.45 d	20.85 e	57.20 f	59.97 c	52.92 c	50.75 a	30.37 e	28.89 f	22.63 d	23.42 d	79.05 f	74.39 f
N3	31.35 c	26.47 d	24.30 c	23.94 d	57.42 f	59.28 cd	52.94 c	51.92 a	28.02 f	26.87 g	21.69 d	24.28 cd	94.35 e	83.37 e
N4	46.05 b	38.98 bc	34.80 b	29.46 c	62.18 b	64.44 a	52.82 c	51.69 a	35.90 b	36.02 a	27.15 a	27.22 a	109.20 d	87.36 e
N5	46.50 ab	40.55 ab	35.55 b	30.86 bc	60.82 c	63.60 a	51.78 d	50.55 ab	34.39 c	34.38 ab	26.38 ab	26.17 ab	112.50 cd	98.64 c
N6	48.75 ab	41.74 a	39.45 a	34.64 a	59.15 de	62.11 b	50.82 e	49.99 ab	31.01 e	30.16 ef	25.08 bc	25.03 bc	131.85 a	120.67 a
N7	47.25 ab	39.63 b	36.45 b	30.51 bc	58.45 e	59.54 c	49.52 f	46.69 cd	31.35 e	31.32 de	24.13 c	24.16 d	126.90 ab	110.31 b
N8	49.50 a	38.93 bc	38.25 a	32.53 b	47.59 g	58.11 de	47.03 g	47.08 cd	33.24 d	32.35 bc	25.71 ab	27.02ab	124.05 b	95.09 cd
N9	45.90 b	37.67 c	36.30 b	31.12 bc	46.59 h	57.03e	47.37 g	45.35 d	32.62 d	33.58 bc	25.73 ab	27.72 a	117.15 c	89.17 de

NTA: N translocation amount, NTAR: apparent N translocation rate, NTCR: N translocation contribution rate, NIPH: N increment in panicle and hull. Different lowercase letters following data within the same column indicate significant differences among treatments at the 5% probability level (*p* < 0.05).

**Table 9:** Effects of different N regulations on N use efficiency of each treatment.

Treatment	NAPE (%)		NPE (kg·kg^−1^)		NAGE (kg·kg^−1^)		NPFP (kg·kg^−1^)		NRG (kg)	
Year	2023	2024	2023	2024	2023	2024	2023	2024	2023	2024
N1	21.36 g	22.27 f	36.38 a	33.22 a	7.16 e	7.39 g	74.38 a	76.60 a	1.52 e	1.52 d
N2	34.90 f	35.86 e	30.42 b	28.25 b	10.47 cd	10.14 d	46.67 b	47.31 b	1.81 d	1.83 c
N3	37.85 e	38.50 d	29.95 bc	27.83 b	11.26 c	10.71 cd	44.87 b	45.13 c	1.87 d	1.89 c
N4	38.14 e	37.92 d	25.93 d	25.01 d	9.87 d	9.48 e	33.39 d	33.57 g	2.10 c	2.10 b
N5	43.39 d	43.05 c	26.47 d	26.10 cd	11.46 c	11.24 c	34.99 d	35.33 f	2.16 bc	2.15 b
N6	52.50 a	52.60 a	29.83 bc	29.04 b	15.62 a	15.28 a	39.15 c	39.37 d	2.27 b	2.17 b
N7	48.99 b	47.37 b	29.00 c	27.32 bc	14.19 b	12.94 b	37.72 c	37.04 e	2.14 c	2.16 b
N8	45.52 c	43.85 c	23.30 e	21.52 e	10.60 cd	9.44 e	28.69 e	27.98 h	2.45 a	2.47 a
N9	45.14 c	43.64 c	21.58 f	20.25 e	9.68 d	8.80 f	27.77 e	27.34 h	2.52 a	2.52 a

NAPE: N apparent efficiency, NPE: N physiological efficiency, NAGE: N agronomic efficiency, NPFP: N partial factor productivity, NRG: N requirement for 100 kg grains. Different lowercase letters following data within the same column indicate significant differences among treatments at the 5% probability level (*p* < 0.05).

**Table 10:** N application rate and period under different N regulation treatments.

Treatment	Total N Applied (kg ha^−1^)	Basal Fertilizer Amount (kg ha^−1^)	Tillering Fertilizer Amount (kg ha^−1^)	N application at Different Leaf Growth Stages			
				Application Rate (kg ha^−1^)	Applying Time (Leaf Growth Stage)	Application Rate (kg ha^−1^)	Applying Time (Leaf Growth Stage)
N0	0	0	0	0	/	0	/
N1	94	94	0	0	/	0	/
N2	175	94	0	0	/	81	13
N3	189	94	94	0	/	0	/
N4	270	94	94	81	9	0	/
N5	270	94	94	81	11	0	/
N6	270	94	94	0	/	81	13
N7	270	94	94	0	/	81	15
N8	351	94	94	81	9	81	13
N9	351	94	94	81	11	81	13

## Data Availability

The original contributions presented in the study are included in the article, further inquiries can be directed to the corresponding author.
